# Evolutionary and Conservation Genetics

**DOI:** 10.3390/life11111160

**Published:** 2021-10-30

**Authors:** Fabio Scarpa, Marco Casu, Daria Sanna

**Affiliations:** 1 Department of Veterinary Medicine, University of Sassari, 07100 Sassari, Italy; 2 Department of Biomedical Sciences, University of Sassari, 07100 Sassari, Italy

Genetic variation, evolution, and conservation are three strictly interconnected words, but none of them exist without the others, unless wanting to waive a complete and operational sense. These terms are considered the key concepts in evolutionary biology and represent the essence of life on our planet. It must be taken into consideration that, while evolution and genetic variation are variants linked to purely natural aspects, conservation is instead—strictly speaking—an activity undertaken by mankind to balance other impacting human activities that strongly interfere, especially in the past three centuries, with the natural evolution and genetic variability of several animal and vegetal species [1]. 

Genetic diversity is imperative for populations to adapt to environmental changes. From an evolutionary viewpoint, genetic diversity manifests itself through differences in several of the phenotypic characters of organisms, such as the color of eyes, hair and skin in humans, the color and type of banding in the shell of snails, as well as the color of flowers in plants. At a deeper level, proteins, amino acids, and DNA sequences, which are present in every biological entity (from viruses to mammals), represent the first traits involved in variation. Looking at the origin of this molecular variability, nucleotide mutation is the primary source of genetic variation representing the raw material based on which natural selection operates and the development of evolutionary principles is based [2]. 

According to the neutral theory of molecular evolution, stochastic fixation of a genetic mutation is the most important driving force behind nucleotide substitutions. Indeed, the majority of substitutions are attributed to a random fixation of neutral or nearly neutral mutations. According to the neutral theory, only a small minority of mutations become fixed by positive selections [3]. As the environmental change is a continuous process, genetic diversity is necessary for populations to evolve and adapt to such changes. As a consequence, the loss of genetic diversity is usually associated with an overall reduction in reproduction and survival rate. Indeed, the changes in genetic variability can primarily drive the evolutionary pathway of a species when its conservation status is compromised by either natural or–more often–human-mediated stress. In such a context, the discipline of conservation genetics focused on the study of this type of threat. It is based on the application of genetic theories and techniques to evaluate the present level of genetic variation of endangered species in its whole range of distribution (when possible) with the aim to address appropriate conservation plans toward reducing the future loss of genetic variability and the risk of species extinction. Indeed, the long-term goal of conservation genetics is to preserve species as dynamic entities that can cope with environmental changes during the time [1,2,3]. 

Conservation genetics derives from population and evolutionary genetics as well as from the theories of quantitative genetics that are at the basis of domestic controlled reproduction. These theories generally deal with large populations whose genetic constitution is controlled by deterministic factors (i.e., selection coefficients). At present, conservation genetics represent a distinct discipline that focuses on the consequences resulting from the reduction in populations (once numerous and with out-crossing to contracted demographic units where the stochastic factors and inbreeding have a preponderant effect. Conservation genetics obviously involves the use of molecular genetic analyses to elucidate the aspects of the biology of species relevant to the their conservation management. All of the approaches and methods that are used to shed light on conservation genetics issues are based on the neutral theory of molecular evolution [1,2,3]. 

In this Special Issue of Life on Evolutionary and Conservation Genetics, we collected a heterogeneous group of studies that aimed at disentangling different genetics and evolutionary processes. The role of the research in the different fields of Evolutionary Biology is of pivotal importance to increase our knowledge of organisms and the biological phenomena involving them. In such a context, evolutionary genetics lays the foundation for understanding the evolutionary mechanisms that shape the genetic variation of each type of organism (such as bacteria, viruses, plants, and animals, etc.), not only (and not anymore) for the mere appreciation of biodiversity, but also to provide a support for the management and conservation of endangered species or species deemed potentially dangerous to the human health. Therefore, this Special Issue aimed to combine the contributions of researchers working globally in the field of evolutionary genetics/biology, population genetics, molecular taxonomy, and conservation genetics, in order to obtain a bulk of papers that underscore the importance of studying genetic variability in organisms for safeguarding biodiversity, in all ways it can be understood [4,5,6,7,8,9,10,11]. 

Usually, the conservation status of endangered or threatened species is investigated through the means of genetic tools. It is important to note that in this context a striking case that is unfolding dramatically before our very eyes and could end with a quick extinction of a species if it fails to reverse the present trend. We are referring to the fan mussel *Pinna nobilis*—a Mediterranean endemic marine bivalve—that is now experiencing dreadful mass mortality. This Mediterranean flagship species, which is included in a protection regime under the Annex II of the Barcelona Convention (SPA/BD Protocol 1995), the Annex IV of the EU Habitats Directive (European Council Directive 92/43/EEC), and, more recently, in Italy, the Legislative Decree 190/2010, Art. 11 for the Marine Strategy Monitoring Program, has represented the focus of a few molecular surveys in the recent times. This species, after undergoing a severe conservation crisis in the 1980s because of a large decline in most populations due to different anthropogenic activities, is currently facing a new dramatic threat, and its conservation status is heavily compromised, since 2016, when a mass mortality event (MME) began to be observed in almost all the Mediterranean areas. So far, numerous epidemiological studies have been performed [12,13,14,15,16,17,18,19,20,21], although none resulted in conclusive identification of the pathogens responsible for the disease that is reducing the populations of *P. nobilis* to their minimum. The case study of *P. nobilis* is a virtuous example of how evolutionary and conservation genetics are strictly interconnected and have required (and will continue to require) studies based on integrated molecular approaches including the endangered host, its presumptive pathogens, and also sentinel species (i.e., heterologous host species that can be hosts for the same pathogens as the threatened species) [21,22]. Furthermore, evolutionary genetics lays the foundation for understanding the evolutionary mechanisms of etiological agents. Indeed, in order to develop an appropriate conservation plan and perform effective management activities, the understanding of the evolutionary mechanism of etiological agents is also required to provide support for the management of pathogens distribution. Finally, considering that the few genetic studies performed in the pre-epidemic period for *P. nobilis* converged in evidencing a past good health for its populations in the entire Mediterranean region [23,24], in the future it would be useful to provide an extended post-epidemic integrative molecular survey, with the hope that *P. nobilis* can recover from MME both naturally and through targeted restocking programs. Regarding this latter point, there are several supporting examples in which the studies devoted to the appraisal of the genetic variability of a species and its distribution, on a small and large geographic spatial scale, have served to design appropriate conservation plans. As a matter of fact, these plans cannot ignore the correct assessment of the genetic variability of the species to be preserved with the aim to, whenever possible, not interfere with the evolutionary processes that, in time and space, have shaped the variability. An interesting study case that involved the research group of the authors of the present Editorial is represented by the endangered species *Patella ferruginea* which is an endemic gastropod of the Western Mediterranean, listed in Annex II of the Barcelona Convention (SPA/BD Protocol 1995) and Bern Convention (Directive 82/72/EEC), in Annex IV of the EU Habitats Directive (European Council Directive 92/43/EEC), and more recently, in Italy, in the Legislative Decree 190/2010, Art. 11 for the Marine Strategy Monitoring Program. This species started declining during prehistoric times mostly because of anthropogenic pressure. Presently, its geographic distribution is restricted to the north African shoreline, Spain, Sardinia, Corsica, and a few scattered sites in the Tuscan Archipelago and Sicily [25]. Despite the now recognized importance of understanding the genetic variability of certain species to understand their evolutionary processes and enable the prediction of any threats in the short term, until now, only a few genetic studies have been performed on this species [26,27,28,29]. 

Based on this background, in 2017 it was possible to implement a European conservation project (LIFE15 NAT/IT/000771), which, at the end of its execution, successfully led to the restocking of the population of *P. ferruginea* in the Ligurian Sea (Western Mediterranean Sea) starting from offspring reproduced in vitro by donors from the Sardinian Marine Protected Area of Tavolara-Punta Coda Cavallo (Western Mediterranean Sea). Therefore, previous data on the genetic variability of *P. ferruginea*, both globally and in the geographic areas involved in this project, enabled the avoidance of both the introduction of non-native genotypes into wild indigenous populations, and “human-mediated” founder effects (i.e., reduced level of genetic variability in a population developed from a small number of founder individuals). 

When talking about evolution, genetics, and host adaptation, we cannot avoid discussing about viruses, which are biological entities capable of complex strategies modeled by the Darwinian natural selection. Viruses represent the best model to investigate such types of processes, because they are cosmopolitan and constantly mutate, thereby presenting a particularly high evolutionary rate that allows for the appreciation of adaptive variations within very few times/generations. Accordingly, the viruses currently present on earth are very well adapted to circumstances, because only the fittest have survived so far, and also because the process of natural selection favors viruses with little pathogenic power. 

In such a context, it should be pointed that, during the last two years of the global spread of the SARS-CoV-2 pandemic, the entire world has witnessed how evolution and genetics dynamics can become extremely dangerous. Indeed, when evolution and natural selection work on wild populations, favoring speciation and producing new evolutionary species sensu Wiley, 1978 (i.e., single lineage of ancestor-descendant populations which maintains its identity from other lineages and which has its evolutionary tendencies and historical fate), they appear to be a good mechanism in promoting the increase in biodiversity. On the contrary, when these forces work on potential etiological agents such as viruses, they produce new variants (potentially deleterious for the host) that can cause epidemics, undoubtedly making evolution appear to be a “bad” entity.

The Nobel prize winner, Sir Peter Medawar, during his speech at the award ceremony said, “no virus is known to do good. It has been well said that a virus is a piece of bad news wrapped up in protein”. The fact remains that evolution is neither good nor bad, simply unexpected. In addition, changes are random, and almost always result in bad combinations for the virus, which tends to weaken over time. However, random changes produce variations, variations facilitate the exploration of new possibilities, and new possibilities can be dire. 

Viruses are peculiar entities, being acellular microorganisms living as obligate endocellular parasites. They are capable of passing through filters that retain bacteria and represent the best expression of efficacy due to evolutionary mechanisms as they exhibit high-performing capabilities. Indeed, a virus needs to be able to pass from one host to another, to enter into the host cells, to take control of the host cells in order to replicate, as well as to leave from the cell and the host. In such a context, considering that both wild and domestic animals can potentially act as a reservoir for many viruses, the occurrence of spillover represents one of the most dangerous events that can happen for humans. 

One of the most serious problems created by viruses is the possibility of causing zoonoses (i.e., infectious diseases transmitted from animals to humans that can evolve to become efficiently transmissible human-to-human infections). Zoonotic infections, originating in wildlife, can pose a significant threat to the human health [30]. The most recent dangerous example of spillover generating zoonotic infection is represented by the current pandemic of SARS-CoV-2. Similarly to other members of the family Coronaviridae, it has a high mutation rate. Indeed, as well as for all RNA viruses, the lack of a quality control mechanism during replication provokes many copying errors at the level of individual code bases. Molecular surveys performed since the late 2019 have indicated that SARS-CoV-2 originated from a bat-borne virus [31], and the global pandemic represents the first time that the virus was transmitted into humans [32]. In addition, in this case, further surveys evidenced that some people were infected with viral strains with an animal sequence signature, indicating the occurrence of a SARS-CoV-2 spillover back and forth between animals and humans within mink farms [33]. 

In such a context, among the family Coronaviridae, there is a further recently discovered threat belonging to the genus Alphacoronavirus, named SADS-CoV (Swine Acute Diarrhea Syndrome Coronavirus), also known as PEAV (Porcine Enteric Alpha-coronavirus) or SeA-CoV (Swine Enteric Alphacoronavirus). Although it is not a zoonosis, SADS-CoV possesses all characteristics to become a threat for human health, thus requiring constant monitoring [34]. So far, it has led to regional outbreaks in the southern region of China [35], producing the typical symptoms of other swine coronaviruses in infected hosts, such as severe diarrhea, vomiting, and dehydration, with a 35% mortality rate in piglets [36]. In this case, considering the limited geographic area (i.e., Fijian and Guangdong), prevention and control may be relatively easy and, so far, this problem has been handled in an outstanding manner. Accordingly, for this etiological agent, several epidemiological surveys based on a phylodynamic molecular approach (phylodynamics is the study of epidemiological, immunological, and evolutionary processes that can act and potentially interact among each other for shaping viral phylogenies) have been performed to identify the temporal and geographical origins so as to provide hints for the management of further epidemics in the future. Indeed, as it is usually common with all epidemics, also in this case an increasing number of surveys are frequently conducted, as alphacoronaviruses are more likely to perform host-switching than betacoronaviruses. In addition, it should be indicated that recombination events between Alphacoronavirus and Betacoronavirus have already occurred [37], which further demonstrates how viruses in general, and coronaviruses in this case, may be unpredictable and easily become a threat to human health. 

In conclusion, irrespective of the biological entities being considered (bacteria, vi-ruses, plants, animals, etc.) and regardless of the fact they cause extinction, promote biodiversity, or evolve under selective pressure, evolution and genetics involve all levels of the expression of life across our planet.
